# A Pilot Study of Radiotherapy and Local Hyperthermia in Elderly Patients With Muscle-Invasive Bladder Cancers Unfit for Definitive Surgery or Chemoradiotherapy

**DOI:** 10.3389/fonc.2019.00889

**Published:** 2019-09-10

**Authors:** Niloy Ranjan Datta, Emanuel Stutz, Emsad Puric, Brigitte Eberle, Andreas Meister, Dietmar Marder, Olaf Timm, Susanne Rogers, Stephen Wyler, Stephan Bodis

**Affiliations:** ^1^Center for Radiation Oncology KSA-KSB, Kantonsspital Aarau, Aarau, Switzerland; ^2^Department of Urology, Kantonsspital Aarau, Aarau, Switzerland; ^3^Department of Radiation Oncology, University Hospital Zürich, Zurich, Switzerland

**Keywords:** muscle-invasive bladder cancers, radiotherapy, hyperthermia, elderly, comorbidity

## Abstract

**Purpose:** To present the outcomes of a pilot study with hyperthermia (HT) and radiotherapy (RT) in elderly patients of muscle-invasive bladder cancers (MIBC) unfit for surgery or chemoradiotherapy (CTRT).

**Methods:** Sixteen elderly patients with unifocal or multifocal MIBCs received a total dose of 48 Gy/16 fractions/4 weeks or 50 Gy/20 fractions/4 weeks, respectively. HT with a radiofrequency HT unit was delivered once weekly for 60 min before RT and a mean temperature of 41.3°C was attained (maximum temperature 41.1–43.5°C). Local control was assessed using RECIST criteria at 3-monthly intervals by cystoscopy with or without biopsy.

**Results:** The median age, KPS and age-adjusted Charlson comorbidity index were 81 years, 70 and 5, respectively. At median follow-up of 18.5 months (range: 4–65), bladder preservation was 100% with satisfactory function. 11/16 patients (68.7%) had no local and/or distant failure, while isolated local, distant and combined local and distant failures were evident in 2, 2, and 1 patient, respectively. Two local failures were salvaged by TUR-BT resulting in a local control rate of 93.7%. The 5-year cause-specific (CS) local disease free survival (LDFS), disease free survival (DFS), and overall survival (OS) were 64.3, 51.6, and 67.5%, respectively while 5-year non-cause-specific (NCS)-LDFS, NCS-DFS, and NCS-OS were 26.5, 23.2, and 38%, respectively. None of the patients had acute or late grade 3/4 gastrointestinal or genitourinary toxicities.

**Conclusions:** The outcomes from this pilot study indicate that thermoradiotherapy is a feasible therapeutic modality in elderly MIBC patients unfit for surgery or CTRT. HTRT is well-tolerated, allows bladder preservation and function, achieves long-term satisfactory locoregional control and is devoid of significant treatment-related morbidity. This therapeutic approach deserves further evaluation in randomized studies.

## Introduction

Bladder cancer (BC) ranks globally as the ninth commonest cancer with an estimated 0.43 million new cases in 2012 (1). Of all the continents, Europe has the highest incidence of BC (2). Furthermore, there is a gradual demographic shift toward an aging society in Europe with the population of people aged 80 years or above expected to double by 2080 and reach 13% of the whole population (3). A similar trend is anticipated globally (4). These factors compel the medical community to explore appropriate therapies in the geriatric population with cancer, where radical surgical approaches and intensive cytotoxic therapies may be associated with higher risks due to age-related comorbidities and poorer treatment compliance and acceptance.

Although neoadjuvant chemotherapy followed by radical cystectomy plus urinary diversion is regarded as a standard of care in muscle invasive bladder cancers (MIBC) (5), elderly patients with associated comorbidities are often deemed unfit to undergo such radical surgery (6, 7). In addition, some may be unfit for bladder preservation approaches using chemoradiotherapy (CTRT) (8, 9). Radiation therapy (RT) alone for such patients has limited therapeutic benefit, although the use of recent RT techniques using image guided intensity modulated RT has shown to achieve good local control with low toxicity (10–13).

Loco-regional hyperthermia (HT) at 39–43°C is a potent radiosensitizer. The likely mechanism of this thermoradiosensitization is attributed to tumor reoxygenation, selective killing of the radioresistant hypoxic tumor cells and inhibition of radiation-induced DNA damage repair (14–16). HT also shows synergy with a wide range of chemotherapeutic (CT) agents and is also a potential immunomodulator (17, 18). Thus, systematic reviews across a wide range of anatomical sites have consistently shown that thermoradiotherapy (HTRT) improves therapeutic outcomes (19–22). The role of HT in non muscle-invasive BC with intravesical CT has been well documented (23–26) and similar efficacy of HT with RT and/or CT has also been reported for MIBC (27–30).

Based on the above thermoradiobiological rationale and the need for a viable treatment option for the rapidly expanding geriatric population unfit for definitive surgery or CTRT, there is a genuine mandate for exploring bladder-sparing strategies in this patient subgroup (31). This report presents the preliminary results of a pilot study in elderly patients with MIBC who due to their age and comorbid conditions or refusal to surgery or CTRT could not be included in an ongoing study with HT and CTRT. They were therefore treated “off-protocol” with HTRT alone and outcomes were reviewed retrospectively.

## Materials and Methods

### Patient Selection

Between December 2012 and February 2018, 17 consecutive patients with MIBC and comorbidities rendering them unfit for radical surgery or CTRT (*n* = 15), or who refused these interventions (*n* = 2), were considered for bladder preserving treatment using HT along with RT. All these patients were considered originally for an ongoing study protocol comprising of CTRT and HT in MIBC. The study protocol was approved by Kantonale Ethikkommission, Aarau, Switzerland and was treated in accordance with the Declaration of Helsinki. However, as all these patients on further investigation were found to be unfit or had refused CT, they were considered for RT and HT alone. All patients gave written consent for treatment with HTRT. Of the 17 patients, one patient with diabetes mellitus developed pyelonephritis and acute cystitis after five fractions of RT and one HT session. She was withdrawn from treatment and has thus been excluded from this analysis. This report is thus an analysis of the outcome of 16 patients with MIBC treated in this pilot study with HTRT.

A detailed medical work-up consisting of hematology and serum biochemistry profiles, creatinine clearance, urine cytology, uroflowmetry, post void residual urine, cystoscopy, contrast-enhanced tomography (CECT) of the thorax, and CECT or magnetic resonance imaging of the abdomen and pelvis were carried out. All patients underwent transurethral resection of the bladder tumor (TUR-BT) with tumor mapping under general anesthesia. Maximal cystoscopic tumor resection was performed and the resected specimens were evaluated by a board-certified histopathologist. All patients were jointly reviewed at the multidisciplinary urology tumor board and endorsed for HTRT alone. The results presented are updated as of November 1, 2018.

### Radiation Therapy

**Figure 1:**
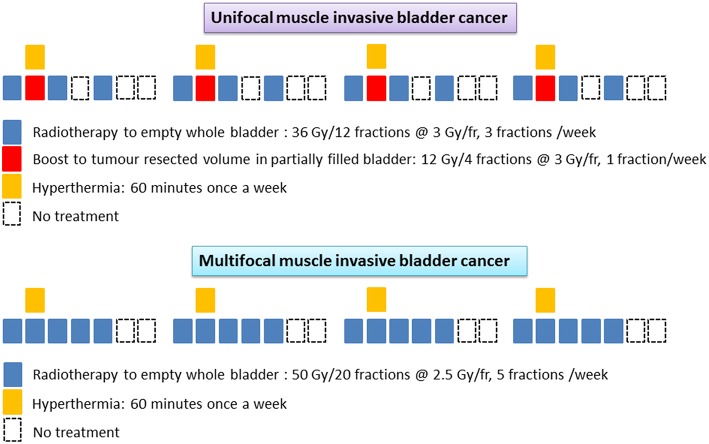
Schematic representation of the radiotherapy and hyperthermia schedules in unifocal and multifocal muscle invasive bladder cancers.

Target volumes were delineated on the RT treatment planning computed tomography (CT) scans. Patients were treated in a supine position supported by individualized patient molds (VacFix vacuum cushions, Par Scientific A/S, Denmark) with minimum 6 MV photons and a 3D-conformal technique. In multifocal tumors, the entire empty bladder wall along with proximal urethra, prostate, and prostatic urethra (in men) were included. The clinical target volume (CTV) of the entire bladder was expanded by 2 cm both laterally and antero-posteriorly and 1.5 cm cranio-caudally. Planning target volume (PTV) was expanded in all directions by 5 mm beyond the CTV. A dose of 50 Gy/20 fractions was delivered over 4 weeks. For unifocal lesions, the resected tumor bed in a partially filled bladder was delineated with local lipiodol infiltration. The empty bladder was irradiated to 36 Gy/12 fractions, three times a week. A boost to the demarcated tumor volume in a partially filled bladder was delivered concomitantly once weekly to a dose of 12 Gy/4 fractions (Figure 1). The CTV was expanded by 5 mm to create the PTV as for multifocal tumors. Electronic portal images (or cone-beam CT) for all fields were taken during the first three fractions and then weekly.

The total duration of treatment was 4 weeks and the estimated biological equivalent dose assuming tumor α/β = 10 Gy, was 62.4 Gy_10_ and 62.5 Gy_10_ for unifocal and multifocal tumors, respectively. No attempt was made to treat the entire pelvic lymph nodes in order to minimize the radiation induced morbidity in these elderly frail patients. All patients were carefully monitored during treatment and any RT dose modifications were performed according to the individual patient's tolerance and compliance to treatment.

### Hyperthermia

**Figure 2:**
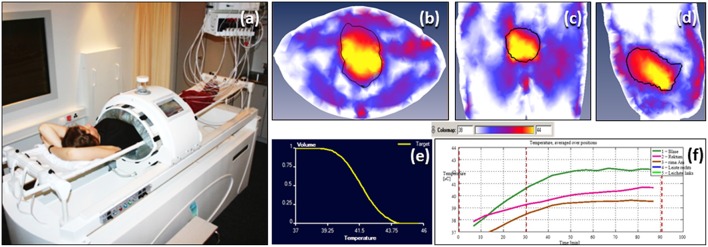
Hyperthermia treatment for bladder cancers **(a)** Patient on the hammock in the deep hyperthermia unit. Temperature distribution in bladder generated on hyperthermia planning system (HYPERPLAN) in **(b)** transverse **(c)** coronal and **(d)** sagittal sections **(e)** cumulative temperature dose volume histogram for bladder **(f)** online temperature recording during hyperthermia.

Deep hyperthermia was delivered using a BSD-2000 with Sigma-60 or Sigma-Eye phased array applicator (Pyrexar Medical, USA, formerly BSD Medical Corporation, USA) in accordance with the European Society of Hyperthermic Oncology (ESHO) quality guidelines (32, 33). A CT scan was carried out in the HT treatment position with full bladder and the treatment volume was based on the GTV drawn on RT treatment plans. An HT plan was generated using Sigma HyperPlan software (Dr. Sennewald Medizintechnik GmbH, Munich, Germany) by segmentation and creation of a grid model of the various body tissues according to their dielectric properties (e.g., tumor, muscle, bone, fat) followed by simulation of the electric fields. Using appropriate power and steering parameters, a specific absorption rate distribution in the target volume was generated using finite element modeling. The resultant specific absorption rate distribution and the tumor temperature were evaluated throughout the target volume (Figure 2). A thermal dose-volume histogram was generated to evaluate the temperature distribution in the tumor and the other adjacent normal tissues.

Hyperthermia consisted of 30 minutes of pre-heating followed by 60 min of HT treatment. Temperature readings were taken every 10 s in the bladder, rectum, groins, gluteal fold, and vagina (in females). Online temperatures were monitored on the skin and the HT device had a circulating water cooling system to keep the skin cooled within tolerance limits. Temperature mapping was carried out along the length of the catheter (typically 16 cm long) every 5–10 min by a flexible multisensor thermometry probe guided through the indwelling Foley's catheter inserted during the HT sessions. The catheter and the temperature probes were removed after each HT session. Systemic body temperature, pulse rate, oxygen saturation, and blood pressure were continuously monitored during HT. The power settings were set not to exceed a temperature of 43°C in the bladder, rectum or vagina and were also adjusted according to the patient's tolerance to HT during the procedure. HT was delivered once weekly and RT within 15–20 min of completion of HT.

### Response Evaluation and Toxicity Scoring

Patients were monitored for acute and late morbidities both during and after the completion of treatment. These included skin and soft tissue, urological, and gastrointestinal adverse effects and were recorded as per the Common Terminology Criteria for Adverse Events (CTCAE), v4.03 (34).

Patients were followed up 6 weeks following the completion of treatment and then every 3 months for the first 2 years and every 6 months thereafter. Cystoscopy with or without biopsies were performed at each follow-up. Local tumor control in the bladder was evaluated by urine cytology and cystoscopy with biopsies and scored as per the RECIST criteria v1.1 (35). A tumor was considered to be “locally controlled” if there was no evidence of demonstrable tumor on cystoscopy and the biopsies were negative for malignancy. Patients with persistent tumor or carcinoma *in situ* were considered to have failed treatment. Radiological investigations, namely CT/MRI/PET scans, were carried out as indicated during follow-up.

Regarding bladder function following treatment, patient assessments were carried out as “patient reported outcomes” focusing on the symptoms of urinary incontinence, urgency, day time frequency, nocturia, urinary stream, and any feeling of incomplete emptying. Patients were specifically asked if they felt satisfied with their bladder function. Those who did not report any problems in any of the above symptoms and expressed satisfaction with their bladder function were considered to be “satisfied.”

### Statistical Evaluation

For survival estimates, the duration of survival was computed from the day of start of treatment till the last follow-up or death. Patients with no evidence of recurrence in the bladder as evident on cystoscopy with or without biopsy were considered free of local disease. Those with no evidence of loco-regional and distant disease as evident on cystoscopy with or without biopsy and on radiological studies were considered as disease-free.

Survival endpoints—local disease free survival (LDFS), disease free survival (DFS), and overall survival (OS), both cause specific (CS) (related to MIBC) and non-cause-specific survival (NCS) were computed using Kaplan-Meier survival estimator. For CS-LDFS, CS-DFS, and CS-OS, patients who died due to causes unrelated to their bladder cancers were considered as “censored.” For NCS-LDFS, NCS-DFS, and NCS-OS, deaths due to any cause were counted as “events.”

Univariate or multivariate analysis using Cox's proportional hazard model for identifying predictors of survival endpoints could not be performed due to low number of cause-specific events for LDFS, DFS, and OS.

All statistical tests were two-tailed and *p*-values ≤ 0.05 were considered significant. Computations were carried out using SPSS version 24.0.

## Results

### Patient Demographics

The median patient age was 81 years (range 52–88) and the median age adjusted Charlson comorbidity index(CCI) was 5 (Table 1) (36). One young patient aged 52 years refused surgery and CTRT and was therefore offered HTRT. The Karnofsky performance status (KPS) ranged from 60 to 90 with a median of 70. Most of the patients (14/16) had a microscopically negative margin following TUR-BT. The majority of patients had T_2_ tumors (12/16) whilst two patients had positive pelvic lymph nodes on pre-treatment evaluation. Two patients (aged 88 and 82 years), staged as T1N0, grade 3 but with poor KPS (70 and 80) and high age adjusted CCI (9 and 4) were treated with HTRT. One of them had a R_2_ pretreatment TURBT resection. Two patients were node positive. Of these one had an 8 mm pararectal node within the treatment portal. The treatment portals were not enlarged to cover the subclinical disease in regional lymphatics. In the second case, the patient had a subdiaphragmatic lymph node which was not reported in the initial CT scan. However, on follow-up, this node was evident. Six patients had unifocal while 10 had multifocal tumors. Histopathologically, all patients had a grade 3 urothelial malignancy.

**Table 1:** Pre-treatment patient characteristics (*n* = 16).

**Parameter**	**Subgroups**	**Value**
Age		52–88 years (median : 81)
Sex	Male: female	15 : 1
Charlson comorbidity index (CCI)	0 : 1 : 2 : 3 : 4 : 5	3 : 5 : 2 : 2 : 3 : 1 (median : 1.5)
Age adjusted CCI	1 : 4 : 5 : 6 : 7 : 8 : 9	1 : 5 : 3 : 3 : 1 : 2 : 1 (median : 5)
KPS	60 : 70 : 80 : 90	3 : 7 : 4 : 2 (median : 70)
Pre-treatment TURB status	R_0_ : R_+ve_	14 : 2
Tumor (T) stage	T1 : T2 : T3 : T4	2 : 12 : 1 : 1
Nodal (N) stage	N0 : N+	14 : 2
M stage	M0	16
Lesions	Single : multiple	6 : 10
Histology	Urothelial	16

*CCI, Charlson comorbidity index; KPS, Karnofsky performance status*.

### Treatment

In 16 patients, the total RT dose varied from 48 to 50 Gy (mean ± SD: 49.2 ± 1.0) and all patients completed the prescribed treatment. The majority received 4 HT sessions at weekly intervals and the mean intravesical temperature was 41.3°C (SD: ±0.7) while the mean maximum temperature was 42.4°C (SD: ±0.7). The distribution of RT and HT parameters for the unifocal (*n* = 6) and multifocal (*n* = 10) tumors are given in Table 2. The median overall treatment time for HTRT was 26 days (range: 25–40).

**Table 2:** Radiotherapy and hyperthermia treatment characteristics for unifocal and multifocal tumors.

**Treatment parameters**	**Unifocal tumors (*n* = 6) (mean ± SD)**	**Mutifocal tumors (*n* = 10) (mean ± SD)**
Radiotherapy dose to pelvis (Gy)	36.0 ± 0.0	50.0 ± 0.0
Radiotherapy dose as tumor boost (Gy)	12.0 ± 0.0	NIL
Total radiotherapy dose (Gy)	48.0 ± 0.0	50.0 ± 0.0
Total hyperthermia sessions (Nos.)	4.0 ± 0.0	4.2 ± 0.4
Mean intravesical temperature (°C)	41.0 ± 1.0	41.5 ± 0.5
Maximum intravesical temperature (°C)	42.3 ± 1.0	42.5 ± 0.6

### Loco-Regional Response

All patients had a complete response (CR) with functional bladder preservation. During follow-up, three patients recurred locally at 10, 17, and 19 months following HTRT. Two patients with isolated local recurrence (pTa and pTis) underwent a repeat TUR-BT and are alive and asymptomatic. The third patient developed local, locoregional and distant failure at 17 months following HTRT. He succumbed to his progressive disease. Thus, the overall local control in the bladder including salvage TUR-BT (in 2 patients) was achieved in 15 out of the 16 patients.

### Distant Failure

Three patients failed at distant sites at 8, 15, and 17 months of completion of HTRT. Of these, one failed both locally and distantly and succumbed to the disease. Of the two patients who failed only distantly, one has died. The other (at diagnosis, age: 78 years; KPS: 90; age adjusted CCI: 4) with metastasis in the spleen and pancreas underwent splenectomy with pancreaticodudenectomy. She had refused to undergo cystectomy or CT for her MIBC and hence was considered for HTRT. Later, at 15 months following HTRT, she was detected to have metastasis in spleen and pancreas. She was advised surgery and willingly underwent splenectomy and pancreaticodudenectomy. Presently the patient is alive (overall survival 46 months) and is locally free of disease in the bladder.

### Survival

The median follow-up is 18.5 months (range: 4–65 months). Survival estimates were computed both as cause-specific (CS) and non-cause-specific (NCS) for LDFS, DFS, and OS.

**Figure 3:**
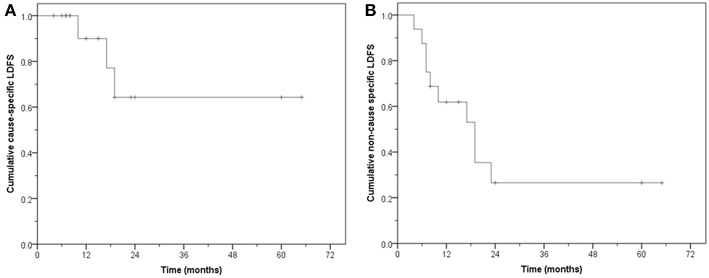
Local disease free survival (LDFS). **(A)** Cause-specific LDFS. **(B)** Non-cause specific LDFS.

Thirteen patients (81.2%) had no local failure on follow-up. Six are alive and seven have died. The CS-LDFS at 1, 2, and 5 years was 90, 64.3, and 64.3%, respectively (median: not reached, mean: 47.4 months, 95% CI: 31.2–63.5) whereas the NCS-LDFS at 1, 2, and 5 years was 61.9, 26.5, and 26.5%, respectively (median: 19 months, 95% CI: 9.9–28.0; mean: 26.8 months, 95% CI: 13.9–39.7) (Figure 3).

**Figure 4:**
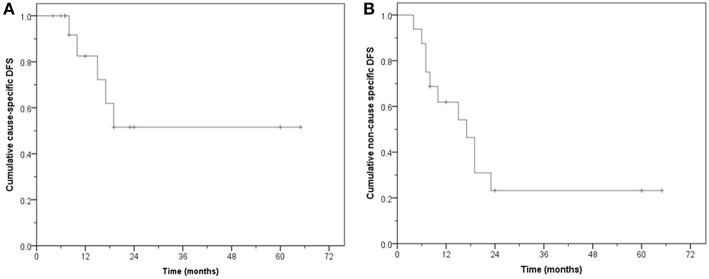
Disease free survival (DFS). **(A)** Cause-specific DFS. **(B)** Non-cause specific DFS.

Of the 16 patients, 11 remain free of disease (68.7%) during follow-up. Five are alive and six have died without evidence of bladder cancer. Five failed either locally and/or at distant sites. The CS-DFS at 1, 2, and 5 years was 82.5, 51.6, and 51.6%, respectively (median: not reached, mean: 40.3 months, 95% CI: 24.6–56.0), while the NCS-DFS at 1, 2, and 5 years were 61.9, 23.2, and 23.2%, respectively (median: 17 months, 95% CI: 9.4–24.5 months; mean: 24.9 months, 95% CI: 13.0–36.8 months) (Figure 4).

**Figure 5:**
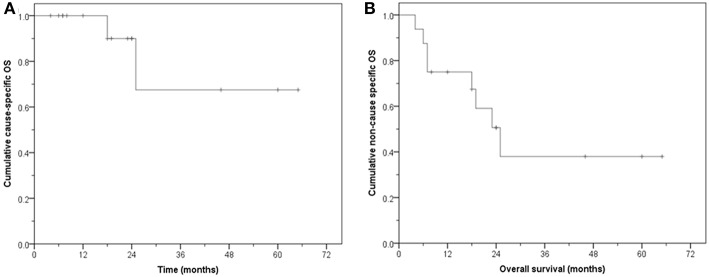
Overall survival (OS). **(A)** Cause-specific OS. **(B)** Non-cause specific OS.

The median follow-up for surviving patients is 24 months (8–65 months). Eight patients are alive, five with no evidence of disease. Of the eight who have died, six had no disease while 2 have died from bladder cancer. Three are alive with evidence of disease. The CS-OS at 1, 2, and 5 years was 100, 90, and 67.5%, respectively (median: not reached, mean: 51.3 months, 95% CI: 34.5–68.0). The NCS-OS at 1, 2, and 5 years was 75, 50.6, and 38%, respectively (median: 25 months, 95% CI: 16.6–33.4); mean: 34.2 months, 95% CI: 20.0–48.4 months) (Figure 5).

The substantial difference between the CS-OS and NCS-OS of LDFS, DFS and OS further highlights that a large proportion of these aged patients died due to non-MIBC causes even though they remained free of their primary tumor.

### Toxicity

All patients complied with HTRT and tolerated the treatment fairly well. Seventy five percent completed treatment within 28 days. One patient had a prolonged overall treatment time of 40 days and had to be hospitalized for problems due to inadvertent self-medication. The acute and late skin, gastrointestinal and genitourinary morbidities were limited to grade 2 or less (Table 3). All patients reported a satisfactory bladder function with no significant symptoms of urinary incontinence, urgency, day time frequency, nocturia, urinary stream, and any feeling of incomplete emptying.

**Table 3:** Acute and late toxicities as per CTCAE v4.0.

**Toxicity**	**Grade**	**Number**
Acute skin toxicity	0 : 1 : 2	1 : 12 : 3
Acute genitourinary toxicity	0 : 1 : 2	3 : 6 : 7
Acute gastrointestinal toxicity	0 : 1 : 2	2 : 3 : 11
Late genitourinary toxicity	0 : 1 : 2	10 : 5 : 1
Late gastrointestinal toxicity	0 : 1 : 2	14 : 1 : 1

*No patient reported with any late skin toxicities*.

## Discussion

Neoadjuvant chemotherapy followed by radical cystectomy with bilateral pelvic lymph node dissection has been the standard therapeutic option in MIBC (5). This has been reaffirmed in the updated 2016 European Association of Urology guidelines for all patients who are fit and willing to undergo surgery (7). For those offered bladder preservation strategies, either due to patient choice or age and associated comorbid conditions, multimodality therapy with CTRT has been recommended as an alternative approach. However, the guidelines make a strong argument against using RT or CT alone as a bladder-sparing treatment strategy in MIBC. A high proportion of patients aged over 70 years continue to receive palliative therapy resulting in poorer outcomes and quality of life (8, 11, 37). This justifies exploring alternative strategies in such patients.

With modern RT techniques, RT treatment planning and its delivery under online image guidance, the major late morbidities to the bladder and bowel have been reduced to <5% (7, 12, 38). This has allowed the exploration of various RT strategies e.g., hypofractionated RT. Turgeon et al. (11) reported their preliminary results using hypofractionated intensity modulated RT (IMRT) with concomitant weekly CT in elderly patients with MIBC (median: 79, range: 72–88). Hypofractionated IMRT at 50 Gy/20 fractions/4 weeks along with weekly gemcitabine 100 mg/m^2^ or cisplatin 40 mg/m^2^ was advocated in 24 patients with MIBC. 3-year overall and cancer-specific survival of 61 and 71% were reported. Seventy five percent of surviving patients had a disease-free functioning bladder. However, the incidence of grade 3/4 acute gastrointestinal, genitourinary, hematological or hepatic toxicities was 29.1%. Thus, adding concomitant CT to RT in a bid to improve outcomes in these elderly patients is associated with higher morbidities.

Hafeez et al. (39) reported their outcomes in elderly MIBC patients using hypofractionated RT alone delivered as 36 Gy in 6 weekly fractions. The cumulative incidence of local progression at 1- and 2- years were 7 and 17% whilst the OS at 1 year was 63%. Acute grade 3 genitourinary and gastrointestinal toxicities were seen in 18 and 4% patients. Grade 3 or higher late toxicity (any) was reported as 6.5% at 6 months and 4.3% at 12 months.

In a recently reported phase I study (40), pembrolizumab, an immune checkpoint inhibitor was added to hypofractionated RT alone delivered as 36 Gy in 6 weekly fractions. Of the 5 patients enrolled in this study, 3 patients developed grade 3 urinary toxicities, 2 of which were attributable to the therapy. In addition, one patient developed a grade 4 rectal perforation. In view of the significant toxicity, the study was stopped and the authors have advised caution when combining hypofractionated RT with immune checkpoint inhibitors in bladder cancers.

HT, with its potent radiosensitizing and synergistic action with a host of CT agents, has been used in both non-invasive bladder cancers and MIBC and been shown to improve outcomes (14, 15, 18, 25, 30, 41, 42). In a prospective randomized trial by the Dutch Deep Hyperthermia group, HTRT significantly improved the complete response rate (CR) over RT alone (73 vs. 51%, *p* = 0.01) (27). However, the 3-year OS was not significantly different in HTRT vs. RT (28 vs. 22%, *p*: not significant). The likely reasons could be that nearly half of the patients had T4 stage tumors. Furthermore, the median overall treatment time was 48 days as RT was delivered at 2Gy/fr to a total dose of 66–70 Gy.

Wittlinger et al. (28) reported the use of HTCTRT in 45 high risk T1 and T2 bladder tumors with a median age of 67 years (range 38–82). Weekly HT was delivered along with 50.4 Gy pelvic RT followed by a 5.4–9 Gy boost and 2 cycles of cisplatin and 5-fluorouracil. Ninety six percent of the patients achieved CR and the 3-year LDFS, CS-DFS, and OS were 85, 88, and 80%. However, acute and late grade III/IV acute toxicities even in this relatively younger aged patient population were 29 and 24%, respectively. Thus, in spite of the impressive results of this study, the toxicity of CT added to HTRT would be poorly tolerated by elderly frail patients.

The present pilot study was conducted to examine the safety and efficacy of treating elderly patients with associated comorbidities with HTRT in an overall treatment time of 4 weeks. Even with low patient numbers, the results indicate that HTRT is both feasible, effective and without any significant treatment related morbidity.

Patients in this age group with comorbidities are likely to die of natural causes. This was evident in this cohort, where six patients (37.5%) died of causes unrelated to MIBC. Two patients with distant metastases succumbed to the disease. One of them was locally controlled and had only distant failure. All patients had a reasonable quality of life with satisfactory bladder function. There were no significant symptoms of urinary incontinence, urgency, day time frequency, nocturia, urinary stream, and any feeling of incomplete emptying. Thus, it is imperative that the therapeutic strategy for patients in this age group needs to be delicately balanced between the intensity of intervention and the treatment-induced morbidity as this could compound the pre-existing comorbidities. Despite its multifaceted therapeutic actions, HT is known to be safe and does not significantly enhance either acute or late toxicities as evident from results in other tumor locations (18, 20–22, 43).

The outcomes from this pilot study indicate that mild to moderate HT along with RT is an efficacious and safe alternative bladder-preservation strategy in geriatric patients with co-morbidities. Globally, few centers currently have HT facilities and a referral pattern could be set up by each region to pool such patients for HTRT. The encouraging results of this pilot study of HTRT in geriatric MIBC patients with associated co-morbidities could be further examined in a randomized multi-centric trial.

## Data Availability

Requests to access the datasets should be directed to niloyranjan.datta@ksa.ch.

## Ethics Statement

This study protocol was approved by Kantonale Ethikkommission, Aarau, Switzerland and were treated in accordance with the Declaration of Helsinki. All patients gave written consent for getting treated with HTRT.

## Author Contributions

ND, ES, EP, BE, and AM drafted the manuscript and worked on the conception, design, and interpretation of the data. ND, ES, EP, and BE organized the database. ND carried out the statistical analysis. ND, ES, EP, BE, AM, DM, OT, SR, SW, and SB reviewed the data analysis and study conclusions. All authors read and approved the final version of the manuscript.

### Conflict of Interest Statement

The authors declare that the research was conducted in the absence of any commercial or financial relationships that could be construed as a potential conflict of interest.
